# The relationship between weight self-stigma, depression and loneliness in people with obesity

**DOI:** 10.4314/ahs.v23i3.80

**Published:** 2023-09

**Authors:** Aysel A Özdemir, Hilal Türkben

**Affiliations:** 1 Department of Nursing, Faculty of Health Sciences, Turgut Özal University Malatya/ Turkey; 2 Department of Nursing, Seydişehir Kamil Akkanat Faculty of Health Sciences Necmettin Erbakan University Konya/Turkey

**Keywords:** Obesity, weight self stigma, depression, loneliness

## Abstract

The research was conducted to determine the level of weight self-stigma, depression loneliness and whether is there a relationship between them. This was a descriptive correlational study. The study was conducted in a diet outpatient clinic of a university hospital. Participants had moderate weight self-stigma, depression and loneliness. Weight self-stigma sub-dimensions self-devaluation (β=0.28; p<0.001) and fear of enacted stigma (β=0.28; p<0.001) equally predicted depression. Fear of enacted stigma predicted social loneliness negatively (β=-.44; p<0.001). Fear of enacted stigma (β =.16, p<.005) and depression (β =.44, p<.001) predicted emotional loneliness positively. Weight self-stigma was positively correlated with depression and loneliness (p<0.001).

## Introduction

The increasing prevalence of obesity is a significant public health concern with physiological, psychological, and social aspects (Bernard et al. 2019). In 2016, 39% of adults over 18 were overweight, and 13% were obese (World Health Organization, 2018). In Turkey, the prevalence of obesity in people over 15 years was 21.1% in 2019 (Turkey Health Research Report 2019).

Obesity is associated with laziness, unhealthy lifestyle choices, and lack of self-control (Brownell et al 2010; Puhl et al, 2015; Schvey et al 2014) but also among the causes of obesity, there are psychological pathologies as well as endocrine, neurological, genetic and sociocultural factors (Sucaklı 2015) which can cause people with obesity to self-stigmatize. Research shows that the weight self-stigma is associated with being teased and bullied, increased anxiety and depression, and decreased physical activity (Wong et al., 2018; Cheng et al 2019; Lin et al., 2018)

Depression is the cause or the result of obesity. Depression is one of the psychological factors affecting eating attitudes and causing an increase in the Body Mass Index (BMI) (Öztürk et al 2018). People with depression are more likely to have impaired eating attitudes and high BMIs (Goldschmidt 2014). People with higher BMIs have more depressive symptoms and higher eating attitude scores than healthy individuals (Annagür 2012). There is a correlation between obesity and depression severity (Öztürk et al 2018).

People with weight stigma and depression are more prone to loneliness. People with obesity face prejudice and ridicule in society (Rubino et al 2020). There are studies reporting that obesity is associated with loneliness (Phelan et al., 2015; Hajek et al., 2019; Hajek 2021). Weight self-stigma and related depression and loneliness may be closely related to the psychopathological dimension of obesity. Nurses have a key role in regulating the nutrition of obese individuals, increasing their physical activity, and providing the necessary care for them to return to a healthy weight. The research was conducted to determine the level of weight self-stigma, depression, loneliness and whether is there a relationship between weight self stigma depression and loneliness in people with obesity. Researchers first thought that depression and loneliness would play a role in weight self-stigma among obese individuals. In this context, it was aimed to examine the effects of depression and loneliness levels on weight self-stigma of people with obesity, with a structural equation model. For this purpose, the concepts of weight self-stigma, depression and loneliness were modelled and tested on the relevant literature. The research hypotheses are;

H 1: There is a significant positive relationship between weight self-stigma and depression among people with obesity.

H 2: There is a significant positive relationship between weight self-stigma and loneliness among people with obesity.

H 3: There is a significant positive relationship between depression and loneliness among people with obesity.

H4: Depression predicts weight self-stigma among people with obesity.

H5: Loneliness predicts weight self-stigma among people with obesity.

## Material and Method

### Research Type

This was a descriptive correlational study.

### Research Setting

The study was conducted in a diet outpatient clinic of a university hospital.

### Population

The study population consisted of all people with obesity admitted to the diet outpatient clinic of the university hospital.

### Sample

Two hundred and fifty people with obesity are admitted to the diet outpatient clinic. The power analysis revealed that a sample of 203 would be large enough to detect significant differences (0.05 confidence interval, 0.30 effect size, and 0.95 sample representativeness). The sample consisted of 219 people who met the inclusion criteria and agreed to participate in the study.

### Inclusion Criteria

* Being open to communication and cooperation.

*Being 18 years or older

* Having a BMI over 30.

### Exclusion Criteria

* Having a history of psychiatric disorder according to self report of participants.

### Data Collection Method and Tools

The co-researcher collected the data between June 2021 and September 2021 using the face-to-face interview method. The data were collected using a Descriptive Characteristics Questionnaire, the Weight Self-stigma Questionnaire (WSSQ), the Beck Depression Inventory (BDI), and the de Jong Gierveld Loneliness Scale (DJGLS). Before data collection, all participants were informed about the research purpose and procedure. Verbal and written consents was obtained from those who agreed to participate. Data collection took 1-15 minutes. COVID-19 measures were taken during data collection.

### Descriptive Characteristics Questionnaire

The Descriptive Characteristics Questionnaire developed by the researchers consisted of seven items.

### Weight Self-Stigma Questionnaire

The Weight Self-Stigma Questionnaire (WSSQ) was developed by Lillis et al (Lillis et al., 2010) and adapted to Turkish by Sevinçer et al. The instrument consists of 12 items and two subscales: self-devaluation (Items 1-6) and fear of enacted stigma (Items 7-12). No items are reverse scored. The Turkish version of the WSSQ has a Cronbach's alpha of 0.83, while the “self-devaluation” and “fear of enacted stigma” subscales have a Cronbach's alpha of 0.74 and 0.81, respectively. “The items are scored on a five-point Likert-type scale (1=completely disagree to “5=completely agree)”. Total scores are calculated for both subscales and the total scale. Higher scores indicate higher self-stigma (Sevinçer et al., 2016). The WSSQ had a Cronbach's alpha of 0.86 in the present study.

### Beck Depression Inventory (BDI)

The Beck Depression Inventory (BDI) is a self-report rating inventory that can be applied to healthy individuals and psychiatric patient groups. The inventory requires no special training or knowledge of psychopathology. One does not have to be a clinician to administer the BDI. This is why the BDI was the instrument of choice in the present study. The inventory was developed by Beck et al. to measure the severity of depression, monitor the course of treatment, and identify the disease (Beck et al., 1961).

The purpose of the inventory is to determine the degree of depression in numerical terms. The inventory was adapted to Turkish by Hisli (Hisli 1989). The Turkish version has a Cronbach's alpha of 0.81, which was 0.80 in the present study. ‘The inventory consists of 21 items, each of which comprises four statements that describe a range of feelings or experiences. These statements are assigned numerical values from 0 to 3. The total score ranges from 0 to 63. Higher scores indicate higher levels of depression (0-10 = normal, 11-16 = mild mood disturbance, 17-20 = borderline clinical depression, 21-30=moderate depression, 31-40 = severe depression, and >40 = extreme depression)’. Participants choose the statements that best describe their feelings for the past week. Then they add up the score for each of the twenty-one items by counting the number to the right of each question they mark. The Turkish version of the inventory has a cutoff point of 17, above which indicates depression that warrants treatment with 90% accuracy (Hisli 1989). The cut-off point in the present study was also 17.

### De Jong Gierveld Loneliness Scale (DJGLS)

The De Jong Gierveld Loneliness Scale (DJGLS) was developed by De Jong Gierveld and Kamphuis (1985) and adapted to Turkish by Çavdar et al. (2015). ‘The scale consists of 11 items and two subscales: social loneliness (Items 3, 6, 8, 9, and 11) and emotional loneliness (Items 1, 2, 4, 5, 7, and 10). Higher scores indicate higher levels of loneliness. The items are scored on a four-point Likert-type scale. There are no reverse-scored items. The scale has a Cronbach's alpha of 0.873 (Çavdar et al., 2015). In the present study Cronbach's alpha was 0.85.

### Ethical Considerations

The study was approved by the Health Sciences Scientific Research Ethics Committee (09.06.2021/42). Written permission was obtained from the chief physician of the hospital (23.06.2021, E-14567952-900-56051). People with obesity were informed about the research purpose and procedure, and verbal and written consent was obtained from those who agreed to participate. Permission was obtained from the authors who adapted the instruments to Turkish.

### Limitations

The study had two limitations. First, the sample consisted of people with similar socio-cultural characteristics. Second the study was conducted in only one hospital.

### Statistical Analysis

The data were analysed using the Statistical Package for Social Sciences (SPSS, v.22.0) at a significance level of 0.05. Number and percentage were used for descriptive data. Arithmetic mean was used to determine the total scores. Correlation analysis were used to compare scores. Structural equation model was established to evaluate the role of depression and loneliness in obese individuals on their weight self-stigma. This model was analysed using the AMOS 23 V program. The role of depression and loneliness on weight self-stigma on people with obesity was evaluated by looking at the standardized regression coefficients. In order to evaluate the validity of the established model, CMIN/df value, CFI and RMSEA values were examined. While evaluating the model fit, the CMIN/df value between 0 and 3, the CFI value above 0.95 and the RMSEA value below 0.08 indicate that the established model fits well and is acceptable.

## Results

Less than half the participants were between 31 and 43 age (36.1%). The majority of the participants were women (78.1%) and married (75.8%). Less than half the participants had primary school degrees (38.8%). More than half the participants had a moderate income (64.4%) and were unemployed (64.4%). 56.2 % of the participants had a BMI of 30-34.9 kg/m2 (Table 1).

**Table 1:** Socio-demographic and clinical characteristics of the sample (n=219)

Characteristics	n	%
**Age (year)**		
18-30	70	32
31-43	79	36.1
44-56	59	26.9
57-69	11	5

**Gender**		
Male	48	21.9
Female	171	78.1

**Marital Status**		
Married	166	75.8
Single	53	24.2

**Education Level**		
Illiterate	3	1.4
Literate	3	1.4
Primary Education	85	38.8
Secondary Education	57	26
University and over	71	32.4

**Perception of Income Level**		
Low	29	13.2
Moderate	141	64.4
Good	49	22.4

**Working Status**		
Employed	78	35.6
Unemployed	141	64.4

**Body Mass Index**		
30-34.9 kg/m^2^	123	56.2
35-39.9 kg/m^2^	62	28.3
40 and over kg/m^2^	34	15.5

Participants had a mean WSSQ score of 34.14±10.80, indicating moderate weight self-stigma. They had a mean BDI score of 19.00±10.38, indicating moderate levels of depression. They had a mean DJGLS score of 24.17±5.11, indicating moderate levels of loneliness (Table 2).

**Table 2:** The weight self-stigma questionnaire, beck depression scale and De Jong Gierveld loneliness scale of the Obese individuals score means (n=219)

Scale	mMin-Max	Mean±XD
The Weight Self-Stigma Questionnaire	12-60	34.14±10.80
Beck Depression Scale	0-63	19.00±10.38
De Jong Gierveld Loneliness Scale	11-44	24.17±5.11

According to the results of the research, it was determined that there was a statistically positive correlation between the self-devaluation and the fear of enacted stigma (r=.565), depression (r=.441) and emotional loneliness (r=.312) (p<0.001). It was determined that there was a statistically negative correlation between self-devaluation and their social loneliness levels (r= -.198) (p<0.001). Although there is a statistically negative relationship between depression and social loneliness (r=-.230), it was determined that there is a statistically positive relationship between depression and emotional loneliness(r=.513) (p<0.001) (Table 3).

**Table 3:** Results of correlation analyses (n=219)

	Self-devaluation	Fear of enacted stigma	Depression	Social Loneliness	Emotional Loneliness
Self-devaluation	1	.565**	.441**	-.198**	.312**
Fear of enacted stigma	.565**	1	.439**	-.294**	.354**
Depression	.441**	.439**	1	-.230**	.513**
Social Loneliness	-.198**	-.294**	-.230**	1	-.111
Emotional Loneliness	.312**	.354**	.513**	-.111	1

Pearson Correlation

** Correlation is significant at the 0.01 level (2-tailed).

**Figure 1:**
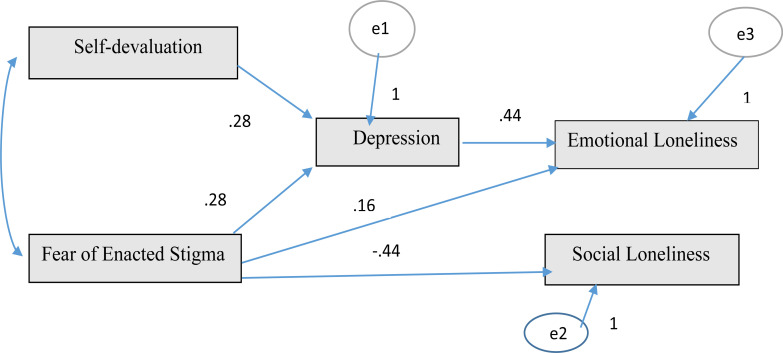
Structural equation modeling

Structural equation modeling was established and tested to determine the role of depression and loneliness levels on weight self-stigma of people with obesity. In the model, depression and loneliness were determined as independent variables and weight self-stigma was dependent variable. The standardized estimation results of the model are presented in Figure 1.

Factor loadings for the latent variables of weight self-stigma scale, self-devaluation and fear of enacted stigma range from 0.45 to 0.88, factor loadings for the latent variable of depression range from 0.30 to 0.84, and factor loadings for the latent variables of social and emotional loneliness range from 0.52 to 0.88. 50% of the explained variance of the weight self-stigma latent variable was calculated from the direct effects of the latent variables of depression and loneliness.

According to the model fit index, the values of x^2^/df=1.031, CMIN/df=3.248, CFI=1.00 and RMSEA=0.01 had acceptable fit values. When the standardized regression (Beta) coefficients were examined, it was found that weight self-stigma had positively predicted depression (β=0.28; p<0.001). Self-devaluation (β=0.28; p<0.001) and fear of enacted stigma sub-dimensions (β=0.28; p<0.001) equally predicted depression. In addition, self-devaluation and fear of enacted stigma explain 24% of the variance in depression.

It was found that the fear of enacted stigma sub-dimension of the weight self-stigma scale had a negative effect on social loneliness. Fear of enacted stigma (β=-.44; p<0.001) predicted social loneliness negatively. Fear of enacted stigma explains 9% of the variance in social loneliness. Fear of enacted stigma (β =.16, p<.005) and depression (β =.44, p<.001) predicted emotional loneliness positively. Fear of enacted stigma and depression explain 28% of the variance in emotional loneliness. Depression and loneliness were effective in explaining the weight self-stigma among people with obesity (Figure 1).

## Discussion

The present study was conducted to investigate the relationship between weight self-stigma, depression and loneliness. Participants had a moderate weight self-stigma, which is consistent with the literature (Jackson & Steptoe, 2018). Participants had a moderate level of depression. Research shows that people with obesity have moderate to high levels of depression (Karagöl et al., 2014; Değirmenci et al., 2015). Obesity is a risk factor for depression (Blasco,et al 2020). Participants had a moderate level of loneliness. Research shows that people with obesity have moderate to high levels of loneliness (Phelan et al., 2015; Yıldırım et al., 2018; Rotenberg 2017). People with obesity who experience weight-based stigma and internalize society's prejudiced attitudes toward weight are more likely to feel isolated and lonely (Phelan et al., 2015). Our results are consistent with the literature.

In the present study, as the level of weight self-stigma of obese individuals increases, their depression levels also increase. People with weight stigma have high levels of depression (Wu & Berry, 2018; Luck-Sikorski et al., 2018). Depression is the cause or result of obesity. Depression is one of the psychological factors affecting eating attitudes and causing an increase in the Body Mass Index (BMI). When faced with stigmatization and exclusion, overweight or obese people are often blamed or blame themselves for not being healthy and thin. Therefore, they may not be happy about their body image and feel ashamed, resulting in depression.

In the present study, while there is a positive relationship between the weight self-stigma and emotional loneliness levels there is a negative relationship between social loneliness levels. It was stated that there was a positive causal effect of BMI on loneliness not including weight bias as a variable (Day et al.., 2018). The fear of enacted stigma predicts social loneliness negatively and emotional loneliness positively. Phelan et al. (2015) also found a correlation between weight self-stigma and loneliness in obese healthcare students. There are also studies that state loneliness can be explained by obesity. Oser et al. found that BMI was associated with loneliness (Oser et al., 2021). Obesity has different consequences in terms of loneliness whereas the end of obesity was not associated with changes in loneliness scores (Hajek & HansHelmut, 2021). Emotional loneliness is related to the lack of an emotional friend whereas social loneliness is related to social isolation and particularly linked to depression (Akgül, 2020). Weight self-stigma scale consists of two sub-dimensions called self-devaluation and fear of enacted stigma. As people with obesity feel worthless and their fear of stigma increases, they may not want to establish emotional intimacy. Therefore, their emotional loneliness may increase. Obese individuals with increased feelings of worthlessness and fear of enacted stigma may become more isolated from social life. In this case, their social relations decrease.

In the present study although there is a negative relationship between depression and social loneliness, there is a positive relationship between depression and emotional loneliness among people with obesity. While depression does not significantly affect social loneliness; explains emotional loneliness at a significant level. Depression predicts emotional loneliness positively. Obesity, loneliness and depression are intertwined spiral expressions. While obesity causes depression, depression also causes obesity. In a recent study higher BMI can be associated with higher levels of depression (Jung et al., 2019). Obese individuals who feel lonely may become prone to depression over time. Loneliness is one of the causes of depression. Our study results are consistent with the literature (Domènech-Abella et al., 2020; Yıldırım et al., 2018).

## Conclusion and recommendations

Depression and loneliness were effective in explaining the weight self-stigma of people with obesity. The results show that the higher the weight self-stigma, the higher the depression and loneliness in people with obesity. Obesity is a significant public health threat that requires weight control. In the treatment of obesity, not only diet or medication or surgery procedures, but also concepts such as loneliness, stigma and depression should be addressed. Therefore, we should identify all physical and mental problems affecting weight control, implement primary, secondary, and tertiary prevention methods, and plan nursing care with a multidisciplinary approach to manage obesity.

## Data Availability

The data that support the findings of this study are available on request from the corresponding author. The data are not publicly available due to privacy or ethical restrictions.
